# The Difficulties and Mental Health Intervention Need of Doctors and Nurses in Biological Emergencies: A Qualitative Study

**DOI:** 10.3389/fpsyt.2021.696823

**Published:** 2021-06-18

**Authors:** Mining Liang, Yamin Li, Qiongni Chen, Haihong Tan, Li He, Caihua Sheng, Yiwen Cai, Xiaojuan Li, Zhanzhou Zhang, Jianjian Wang, Qian Wang, Jincai Guo

**Affiliations:** ^1^Clinical Nursing Teaching and Research Section, The Second Xiangya Hospital, Central South University, Changsha, China; ^2^Mental Health Institute of the Second Xiangya Hospital, Central South University, Changsha, China; ^3^Department of Pharmacy, Changsha Stomatological Hospital, Changsha, China

**Keywords:** biological disaster, 2019 novel coronavirus disease (COVID-19), mental health intervention needs, doctors and nurses, qualitative research

## Abstract

When a biological public health event breaks out, due to the characteristics of their work, doctors and nurses must face risks directly when the situation is unknown. Their difficulties and psychological pressure are unimaginable. However, few studies have investigated the difficulties encountered by those doctors and nurses and their requirements for psychological interventions. This study aimed to explore the difficulties and psychological intervention needs of doctors and nurses during the new biological public health events in China in 2019. We carried out a qualitative study using a phenomenological approach. We used convenience sampling to identify participants who provided direct care and treatment for patients with biological events such as coronavirus disease 2019 (COVID-19). They participated in semi-structured, in-depth face-to-face interviews. The interviews were transcribed and analyzed using Colaizzi's seven-step method. Analysis of this study was divided into the difficulties encountered by doctors and nurses and their mental health need. The difficulties encountered by doctors and nurses included four themes: being worried about the impact on others, lack of knowledge and skills, difficult patients, being socially isolated, and the feeling of uncertainty. The mental health need was summarized into two parts, needs expressed by doctors and nurses and needs observed by researchers. Doctors and nurses mostly did not feel that they needed any psychological support, but the researchers noticed several signs of stress or potential mental health problems among interviewees. Doctors and nurses faced significant complex and multidimensional difficulties. Many denied needing psychological support, even though the researchers noted signs that it might be helpful. Interventions and support strategies that involve mental health promotion activities should consider individual needs related to doctors and nurses' situation.

## Introduction

Biological events that have caused significant mass morbidity, mortality, and fear are not uncommon in human history. They pose a serious threat to the health and safety of citizens and cause huge financial and labor burdens on the affected communities and health systems (1). In December 2019, an outbreak of a novel coronavirus pneumonia occurred, and the virus quickly spread around the world (2, 3). 2019 novel coronavirus disease (COVID-19) is an aspiratory infectious disease (4). The virus is transmitted mainly by respiratory droplets or direct contact with infected patients' body fluids. The World Health Organization declared the novel coronavirus pneumonia outbreak a global pandemic, and large numbers of doctors and nurses around the world are now treating patients with COVID-19 (5).

The largest tertiary hospital in Hunan Province, the Second Xiangya Hospital of Central South University, undertook much of the investigation and treatment of patients with suspected COVID-19. Frontline staff at the hospital needed to carry out tasks while wearing protective clothing, eye protection and other protective equipment, which made the work more difficult. Patients also had different levels of anxiety, anger and hostility as a result of fear of the disease and isolation from society and family. These negative emotions and uncooperative behaviors were difficult for doctors and nurses, and put them under both physical and mental pressure. Previous studies found that 93.5% of doctors and nurses reported difficulties from treating patients with Severe Acute Respiratory Syndrome (SARS), a previous coronavirus disease (6). Doctors and nurses caring for patients with SARS reported fatigue, poor sleep, concerns about their own health, and fear (7). Doctors and nurses' ongoing health and safety are crucial for both patient care and the control of any outbreak (8).

The Mental Health Institute and the Medical Psychological Research Center of the Second Xiangya hospital, and the Chinese Medical and Psychological Disease Clinical Medicine Research Center responded rapidly to the psychological pressures on doctors and nurses. A detailed psychological intervention plan was developed, which covered three main areas. First, there was a psychological intervention medical team, which provided online courses to help doctors and nurses to manage common psychological problems. Second, providers could access one-to-one telephone or tele-video psychological counseling, which provided guidance and supervision to solve psychological problems. Finally, there were also group psychological interventions, which provided activities to release pressure. However, the implementation of these psychological intervention services encountered obstacles, and doctors and nurses were reluctant to participate in the group or individual psychology interventions available.

Previous studies on epidemics of infectious diseases mainly investigated doctors and nurses' emotional experience, preparedness and coping strategies (9). However, there has been little qualitative research examining the difficulties encountered by doctors and nurses and their needs for psychological intervention during the outbreak of biological events. The aim of this study was to use a qualitative and phenomenological approach to identify the difficulties and psychological intervention needs of doctors and nurses. The findings may inform the development and implementation of intervention programs tailored to the psychological needs of doctors and nurses. They may also have important implications for informing psychiatrists and hospital managers and helping them to improve pre-job training, psychological interventions, management programs, and support strategies for doctors and nurses.

## Materials and Methods

### Sample and Participant Selection

We carried out a qualitative study using face-to-face interviews. A phenomenological approach was used to obtain detailed descriptions. Phenomenology is a qualitative research tradition that focuses on exploring how individuals make sense of the world, to provide insightful accounts of their subjective experiences (10). The descriptive phenomenological method is therefore one of the best ways to describe the difficulties and mental health intervention needs during the outbreak of COVID-19 in China.

Convenience sampling was used to identify potential participants. The inclusion criteria were doctors and nurses who provided direct care and treatment for patients with COVID-19. Exclusion criteria were participating in other studies. We continued sampling until we reached data saturation and no new information was emerging from participants' experiences.

This study was approved by the ethical committee of the Second Xiangya Hospital, Central South University (Approval Number: 2020007). All participants were informed that the interview data would only be used in this study and all personal information would be concealed. Verbal informed consent was obtained when participants were first invited to join the study, and formal written informed consent was obtained before the interviews. All participants were informed that they could refuse to answer any question or withdraw from this study at any time.

### Interviews and Procedure

A semi-structured interview guide was developed from the literature and with advice from professionals. With participant permission, all interviews were audio-recorded. Participants' gender, age, years in work, education status, work pattern, position, and the date they started working on the COVID-19 ward were obtained at the start of the interview. They were then asked questions such as “Are you worried about being infected?”, “What difficulties did you encounter when treating patients during the epidemic?”, “What kind of help and support do you want?”, and “Do you need any psychological interventions from the psychology department? Why?”. The data collection process was divided into three steps: (1) identifying potential participants using inclusion and exclusion criteria; (2) inviting them to participate in face-to-face interviews; (3) interviewing participants in the hospital when they were off duty; and conducting formal interviews lasting 30–40 min and involving two researchers. One researcher interviewed, and the second managed the recording and took notes. Both researchers (ML and QC) also recorded participants' non-verbal behavior (facial expressions, tone, and mood). The interviewers were unknown to the participants.

### Analysis Strategy

Two researchers transcribed the interview records into text within 48 h of the interviews. One researcher transcribed, and the other checked the consistency of the text and the records. Colaizzi's seven-step method was used to analyze the data (11):

(1) Transcribing all the participants' words.
(2) Extracting significant statements. The transcripts were carefully read and re-read to ensure the researchers were familiar with all the content. Two researchers separately analyzed the transcribed text and selected meaningful statements from the data.
(3) Creating formulated meanings. Two researchers separately summarized and refined the meanings to develop common characteristics.
(4) Aggregating formulated meanings into theme clusters. Two researchers combined their results to develop themes.
(5) Developing an exhaustive description. All themes were connected back to the interview data to obtain complete descriptions.
(6) Identifying the fundamental structure of the phenomenon. We identified the two pathways to describe the psychological experience of doctors and nurses during the COVID-19 outbreak in China: the difficulties they met and their mental health intervention need.
(7) Returning to participants for validation. The analysis results were returned to the participants for verification and revision.

The data were analyzed in Chinese, and only translated in English when summarizing the results and writing the manuscript. Two researchers chose the example quotations from the database and translated them into English. The draft of the translated data was sent to professors teaching Nursing or English at the university, and the researchers then revised and selected the final data in English.

## Results

In total, 13 doctors and nurses, of whom 10 were nurses and three physicians, were willing to participate and signed an informed consent form after being told about all the procedures involved in the study. All participants cared for patients with COVID-19 in the hospital. Their time working on COVID-19 wards before the interviews ranged from 3 to 14 days. They were still working on the COVID-19 wards at the time of their interviews. Thematic redundancy was achieved with the 13th interview. Participants' demographic characteristics are shown in Table 1.

**Table 1:** Participant information.

**Number**	**Gender**	**Age**	**Work years**	**Education**	**Work patten**	**Position**	**Anti-epidemic work duration**
Nurse 1	Female	35	15	Undergraduate	Shift work	Nurse	3 days
Nurse 2	Female	29	5	Undergraduate	Shift work	Nurse	3 days
Nurse 3	Female	37	16	Undergraduate	Shift work	Nurse	14 days
Nurse 4	Female	35	15	Undergraduate	Shift work	Nurse	3 days
Nurse 5	Female	25	3	Undergraduate	Shift work	Nurse	14 days
Nurse 6	Female	29	7	Undergraduate	Shift work	Nurse	3 days
Nurse 7	Female	37	12	Undergraduate	Shift work	Nurse	3 days
Nurse 8	Female	27	5	Undergraduate	Shift work	Nurse	3 days
Nurse 9	Female	42	21	Undergraduate	Day work	Head Nurse	14 days
Nurse 10	Female	31	8	Undergraduate	Day work	Head Nurse	14 days
Doctor 1	Male	35	6	Doctor	Day work	Doctor	7 days
Doctor 2	Male	26	3	Undergraduate	Shift work	Doctor	3 days
Doctor 3	Male	37	7	Doctor	Day work	Doctor	7 days

The difficulties encountered by doctors and nurses treating patients with COVID-19 included being worried about the impact on others, lack of knowledge and skills, the stress resulting from contact with stressed, anxious and difficult patients, being socially isolated and managing uncertainty. Their mental health intervention needs include both those observed by researchers and those expressed by the doctors and nurses themselves (Table 2).

**Table 2:** Main concepts and subconcepts of the difficulties and mental health intervention need of doctors and nurses during the COVID-19 outbreak.

**Main Categories**	**Subcategories**
Worrying about the impact on others	Worrying about the impact on family
	Worried about the impact on colleagues
Lack of knowledge and skills	Unfamiliar with working with protective equipment
	Unfamiliar with the treatment of infectious diseases
Stress relating to patients	Patient's anxiety and panic The patient does not cooperate with treatment
Social isolation and uncertainty	The need to prevent infection Uncertainty in the outcome of infectious diseases
Needs expressed by doctors and nurses	Need more uninterrupted rest
Needs observed by researchers	There are obvious psychological problems and psychological intervention is needed

### The Difficulties Encountered by Doctors and Nurses

#### Worrying About the Impact on Others

##### Worrying-About-the-Impact-on-Family

Doctors and nurses were afraid of bringing the virus home to their family because of their close contact with patients. Getting infected themselves was not an immediate worry once staff began work.

“I did not worry about this being infected once I started work.” (Nurse 1)

“I do not want my family to worry about me, so I did not tell my family I was on the frontline. I am afraid to talk to my family through video link, and I asked my husband to tell my parents that I have been busy with my work recently and that's why I have had no time to contact them.” (Nurse 2)

“Because I was worried about bringing the virus to my children and parents, I would wait on the road for half an hour before returning home.” (Doctor 3)

“I was very worried when I heard that my colleague's father had a fever and they were both admitted to hospital and isolated.” (Nurse 6).

##### Worried-About-the-Impact-on-Colleagues

For head nurses, the biggest concerns were the fear that colleagues might be infected, and worry about the shortage of protective equipment. How to ensure the safety of doctors and nurses was also a priority. The most important thing was to provide sufficient protective equipment, but this was in short supply early on during the epidemic.

“My biggest concern is to make sure that none of my colleagues are infected by novel coronavirus. I'm not afraid of being infected myself.” (Nurse 10)

“I was anxious when I saw colleagues who were not skilled in using protective equipment.” (Nurse 10)

“When I distributed protective equipment to my colleagues, I felt very nervous because I was worried supplies would run out.” (Nurse 9).

##### Lack of Knowledge and Skills

Doctors and nurses were unfamiliar with some operations and protective equipment. They felt uncomfortable wearing protective equipment and it reduced their ability to function, with the several layers of gloves required. Doctors and nurses who took care for COVID-19 patients, most of them had none or little respiratory or infectious disease working experience before, and in-depth training for these staff was difficult to achieve. Few of them had used the protective equipment before, and were not familiar with isolation and protection technology. Quite a few were not familiar with the treatment of respiratory infectious disease. Some lacked training on ventilator equipment, and did not know how to use advanced instruments such as non-invasive respirators, and extracorporeal membrane oxygenation.

“It is very difficult to give injections to patients when we wear double-layer gloves, and the protective goggles are easy to fog. There are also many physical tasks, such as taking out garbage, caring for them on a daily basis admitted patients, disinfection after patients have been discharged, delivering food and medicine, and giving injections. I was sweaty after wearing the protective clothing for only 2 h.” (Nurse 5)

“Although there was careful training before working, on my first day I was still unfamiliar with the protective clothing. I did not have previous experience working in the infectious diseases department and had rarely used protective equipment.” (Nurse 1)

“I do not have sufficient experience with respiratory infectious diseases because my previous work in the infectious diseases department was mainly focused on liver disease.” (Nurse 5).

##### Stress Relating to Patients

Doctors and nurses did not know how to deal with patients who were unwilling to be quarantined at the hospital or would not cooperate. As panic spread, some patients kept asking doctors and nurses for the results of nucleic acid tests, and some were not willing to be hospitalized and quarantined because they only had mild symptoms. Others were eligible for home quarantine but refused to leave the hospital. Doctors and nurses found it hard to deal with these patients.

“Some patients are unwilling to be quarantined at the hospital. They keep ringing the bell and asking when they can go home.” (Nurse 3)

“One patient did not understand why she had to stay one meter away from her mother. After we explained the reason to her, she was still very angry and unwilling to cooperate. Her mother had come from Wuhan and she felt that it might be discrimination.” (Nurse 4)

“We suggested that patients should isolate at home when their nucleic acid test results were negative, but many were worried the test results were wrong and refused to go home.” (Nurse 7)

“When a patient and their family members took a nucleic acid test at the same time, but the results were due back at different times, some family members became very angry. The young nurses often did not know how to deal with this.” (Nurse 4).

##### Social Isolation and Uncertainty

Doctors and nurses felt lonely because they are separated from colleagues, families and communities. They are also worried about the uncertainty caused by the epidemic.

“We do not communicate with each other when we are off duty.” (Nurse 8)

“It is inconvenient to communicate with colleagues when I am wearing protective clothing and goggles.” (Nurse 5)

“I don't go anywhere because I work in the infectious diseases department.” (Nurse 5)

“I feel all right when I focus on work, but I find it really hard to think about the future.” (Doctor 2).

#### The Mental Health Intervention Needs

##### Needs Expressed by Doctors and Nurses

Many staff were clear that they did not need a psychologist, but just needed more uninterrupted rest.

“My mental health is good. I don't need psychological help.” (Nurse 2)

“I'm fine. Don't worry.” (Doctor 1)

“At present, I don't think we need psychological intervention. I just need more uninterrupted rest.” (Nurse 4).

##### Needs Observed by Researchers

Researchers observed that individual nurses showed excitability, irritability, unwillingness to rest, and signs of psychological distress, although these were denied by those involved. The researchers concluded that at least some doctors and nurses had obvious psychological issues that they did not want to acknowledge.

## Discussion

The difficulties encountered by doctors and nurses caring for patients with biological events included worrying about the impact on others, lack of knowledge and skills, stress from managing difficult patients, being socially isolated and coping with uncertainty. The biggest concern was the impact on others. Biological events have obvious gregariousness due to their infectiousness, such as, COVID-19 is transmitted directly from person to person and shows familial aggregation (12), and therefore the doctors and nurses were worried about taking the virus home to their families from close contact with patients. Son et al. also found that fear of infecting family members was the main experience of healthcare staff involved in treating patients during infectious public health incidents (13). A shortage of protective equipment is also a common difficulty in public health emergencies, which puts doctors and nurses in an unsafe clinical environment and directly threatens their security (14), and this was also found in our study. A previous study found that many doctors and nurses were infected early on in an outbreak of Ebola virus because of an insufficient supply of personal protective equipment and insufficient attention to the process of putting on and taking off personal protective equipment (15). Head nurses were worried about employees being infected because of the limited supply of personal protective equipment, and weak public healthcare infrastructure. Secondly, doctors and nurses felt a lack of knowledge and skills. Bennett et al. found that front-line National Health Service workers were not ready while caring for patients with COVID-19 (16). They were fully prepared and had all volunteered to work on the COVID ward, but still found it difficult to manage because of their lack of experience in this area. Thirdly, doctors and nurses were under stress from dealing with difficult patients. Patients generally believe that hospitals are a safe place for treatment, and should therefore be prepared to respond to their needs (17). However, it was difficult to deal with patients making additional demands that were not consistent with best practice. Lastly, doctors and nurses felt socially isolated and uncertain. Li et al. found that isolation from family and society can directly and indirectly affect the mental health of doctors and nurses (18). The purpose of quarantine is to reduce the risk of infection, not to limit interpersonal contact, which will affect the mental health of doctors and nurses (19). Goh et al. also found that nurses reported physical and psychological challenges relating to working conditions of the hospital in the initial months of the pandemic in Singapore (20).

The mental health intervention needs in this study included both those observed by the researchers and needs expressed by the doctors and nurses. During the early part of the outbreak of biological events, the psychological needs of doctors and nurses were not clear. Staff were also often unwilling to take up psychological interventions. They showed little interest in online psychological courses, and very few took advantage of telephone counseling. There may have been several reasons for this. It is possible that staff were afraid of being stigmatized if they betrayed their “weakness” to others. A general stigma about mental health might also hinder the use of mental health services (21). The Chinese cultural background may make people feel that mental health problems are shameful, and thus they are not willing to accept mental health assistance. Alternatively, the extreme pressure under which staff were working may have prevented them from considering their own emotional experiences and psychological needs. Previous research has also found that the take-up rate for psychological interventions is often low in individuals suffering major traumatic events and people frequently refused to ask for help (22). Maslow's hierarchy of needs suggests that people who experience traumatic events would prioritize their physical safety, not mental health. This might mean that staff were not interested in participating in group interventions focused purely on their mental health needs. Schwarz and Kowalski found that reluctance to use mental health services was a manifestation of avoidance of reminders of the trauma, which was the main symptom of a post-traumatic response. In other words, people avoided services that might mean reliving the experience (21). The lack of willingness of doctors and nurses to seek mental health assistance may also have been related to the difficulty of building trust quickly between doctors and nurses and psychologists during a major pandemic. Both the doctors and nurses and psychologists were brought together quickly and were not familiar with the new working environment. This made it difficult to establish a trusting therapeutic relationship. However, researchers observed that individual nurses showed excitability, irritability, unwillingness to rest, and other signs of psychological distress. Hacimusalar et al. also found that healthcare workers were more affected psychologically in the COVID-19 pandemic compared to the society in Turkey (23), suggesting that some interventions were needed.

We suggest that there may be some interventions that could reduce the difficulties of doctors and nurses. It might be helpful to video doctors and nurses' routines in the hospital to share with their families, and alleviate their concerns about what was happening. The hospital union and other departments could also organize support for family members. Doctors and nurses need to know about both characteristics of infectious diseases and protective equipment, and we suggest that some kind training might be appropriate before starting work. In extreme cases where a patient did not cooperate, ward staff should be encouraged to use hospital security staff to maintain order and protect frontline staff from harm. Staff should also be given regular and accurate updates on the COVID-19 outbreak, to reduce their uncertainty and fear (24). It may not be possible to provide obvious “psychological” interventions from the beginning. However, it might be possible to establish a good therapeutic relationship by using psychologists to help doctors and nurses solve difficulties such as dealing with patients with psychological problems, and arranging training in how to identify psychological problems in patients and other staff. Some psychological intervention programs could be adjusted to meet the needs of doctors and nurses, including through provision of leisure activities such as table tennis, medical rehab exercises and relaxation training, which could help to reduce stress and feelings of loneliness (25). Doctors and nurses could also ask psychologists to work with patients who were difficult to communicate with, or have emotional outbursts. Meanwhile, Arnetz et al. suggested that healthcare institutions should provide opportunities for U.S. nurses to discuss the stress they are experiencing, support one another, and make suggestions for workplace adaptations during this pandemic (26).

This study had some limitations. Firstly, all the participants were from the same hospital, and therefore the results may not be generalizable to other places. Secondly, this study only used interviews to collect data and the participants' answers may have been influenced by the interviewers. However, we tried to rule out this effect. For example, the researchers received training on interviewing techniques, and we used semi-structured open-ended questions to alleviate the bias caused by different ways of asking questions. This study also has some strengths. The first is that we collected data during the outbreak of COVID-19, from staff working on the COVID wards at the time. This avoided any recall bias among participants. The study also involved a range of doctors and nurses, including doctors, nurses and head nurses, ensuring that different perspectives were considered.

### Conclusion

Participants in this study were interviewed during the COVID-19 epidemic. The qualitative design of this study provided a detailed and in-depth understanding of the difficulties they encountered and their psychological needs during the outbreak. The results suggest that doctors and nurses would prefer more uninterrupted rest and better access to protective equipment than psychological interventions. Interventions and support strategies that involve mental health promotion activities may therefore be better, although these should consider the individual needs related to doctors and nurses' situations. This study may provide support to help respond to future unexpected infectious disease outbreaks.

## Data Availability Statement

The original contributions presented in the study are included in the article/supplementary material, further inquiries can be directed to the corresponding author/s.

## Ethics Statement

This study was approved by the ethical committee of the Second Xiangya Hospital, Central South University (Approval Number: 2020007). The patients/participants provided their written informed consent to participate in this study.

## Author Contributions

ML: data curation and writing—original draft preparation. YL, HT, LH, CS, YC, XL, ZZ, and JW: supervision. QC: conceptualization, methodology, and writing—reviewing and editing. QW: original draft preparation. JG: reviewing and editing. All authors contributed to the article and approved the submitted version.

## Conflict of Interest

The authors declare that the research was conducted in the absence of any commercial or financial relationships that could be construed as a potential conflict of interest.
